# Supplementary material for: Nutrient content and stoichiometry of pelagic Sargassum reflects increasing nitrogen availability in the Atlantic Basin
Source: Nat Commun. 2021 May 24;12:3060. doi: 10.1038/s41467-021-23135-7 (PMC8144625; doi:10.1038/s41467-021-23135-7)
Supplement: Supplementary file 1 — Supplementary Information [file 41467_2021_23135_MOESM1_ESM.pdf]

Supplementary Information

Nutrient content and stoichiometry of pelagic *Sargassum* reflects increasing nitrogen availability in the Atlantic Basin

B. E. Lapointe*[1], R. A. Brewton[1], L. W. Herren[1], M. Wang[2], C. Hu[2], D.J. McGillicuddy, Jr.[3], S. Lindell[3], F. J. Hernandez[4], P. L. Morton[5]

[1]Harbor Branch Oceanographic Institute, Florida Atlantic University, 5600 US 1 North, Fort Pierce, FL 34946
[2]University of South Florida, 140 Seventh Ave. South, St. Petersburg, FL 33701
[3]Woods Hole Oceanographic Institution, 86 Water St., Woods Hole, MA 02543
[4]Division of Coastal Sciences, University of Southern Mississippi, 703 East Beach Drive, Ocean Springs, MS, USA 39564
[5]Florida State University/National High Magnetic Field Lab, 1800 E. Paul Dirac Drive, Tallahassee, Florida 32310

*Corresponding author: Brian Lapointe, blapoin1@fau.edu

Supplemental Results

Changes in *Sargassum* tissue chemistry

Sargassum tissue chemistry was statistically similar between *S. fluitans* and *S. natans* for the entire NA dataset. Specifically, *S. fluitans* and *S. natans* had similar elemental composition (MANOVA, Pillai's lambda = 0.006, $F =_{3, 470}$ 1.01, P = 0.388; Supplementary Table 1) and C:N:P molar ratios (MANOVA, Pillai's lambda = 0.005, $F =_{3, 470}$ 0.792, P = 0.499; Supplementary Table 2). For elemental composition, the interactions between species, decade, and season were not significant (Supplementary Table 1). The overall mean values for %C, %N and %P were 28.0 ± 0.15, 1.18 ± 0.02, and 0.11 ± 0.003 (all n = 488). The overall mean molar C:N, C:P, and N:P ratios were 32.7 ± 0.71, 853 ± 23, and 26.6 ± 0.48 (all n = 488). For the multivariate analyses of C:N:P ratios, there was a significant 2-way interaction between species and season (MANOVA, Pillai's lambda = 0.074, $F =_{9, 1416}$ 3.96, P < 0.001) and 3-way interaction between decade, species, and season (MANOVA, Pillai's lambda = 0.0677, $F =_{9, 1416}$ 3.63, P < 0.001). The subsequent univariate ANOVAs did not identify any of the C:N:P ratios to be significant for these interactions (all P > 0.05; Supplementary Table 2). For nutrient ratios, there was a multivariate interaction between decade and season (MANOVA, Pillai's lambda = 0.439, $F =_{9, 1416}$ 2.34, P = 0.013; Supplementary Table 2), however none of the univariate ANOVAs were significant (all P > 0.05; Supplementary Table 2).

Supplementary Table 1: *Sargassum* elemental composition MANOVA Table.
Multivariate analysis of variance (MANOVA) table of tissue elemental composition (%C, %N, %P) from *Sargassum natans* and *S. fluitans* collected throughout the North Atlantic Ocean. Factors included were by decade (1980s vs 2010s), species (*S. natans* vs. *S. fluitans*), Northern Hemisphere meteorological season (winter, spring, summer, and fall), and all interaction effects. Subsequent univariate analysis of variance (ANOVA) are shown for significant factors. Significant (<0.05) *P* values are bold**.**

**Test** / Factor (s)	SS	df	*F*	*P*
**MANOVA**				
Decade	-	3, 470	39.4	**<0.001**
Species	-	3, 470	1.01	0.388
Season	-	9, 1416	8.13	**<0.001**
Decade * Species	-	3, 470	1.32	0.266
Species * Season	-	9, 1416	0.51	0.871
Decade * Season	-	9, 1416	2.95	**0.002**
Decade * Species * Season	-	9, 1416	0.51	0.976
**ANOVA %C**				
Decade	440	1	53.8	**<0.001**
Season	63.3	3	2.58	0.053
Decade * Season	46.5	3	1.90	0.129
**ANOVA %N**				
Decade	0.118	1	5.01	**0.026**
Season	1.20	3	17.1	**<0.001**
Decade * Season	0.212	3	3.00	**0.030**
**ANOVA %P**				
Decade	1.10	1	31.4	**<0.001**
Season	1.76	3	16.7	**<0.001**
Decade * Season	0.410	3	3.91	**0.009**

Supplementary Table 2: *Sargassum* tissue nutrient stoichiometry MANOVA table.
Multivariate analysis of variance (MANOVA) table of tissue nutrient stoichiometry (C:N, N:P, C:P) from *Sargassum natans* and *S. fluitans* collected throughout the North Atlantic Ocean. Factors included were by decade (1980s vs 2010s), species (*S. natans* vs. *S. fluitans*), Northern Hemisphere meteorological season (winter, spring, summer, and fall), and all interaction effects. Subsequent univariate analysis of variance (ANOVA) are shown for significant factors. Significant (<0.05) *P* values are bold.

**Test** / Factor(s)	SS	df	*F*	*P*
**MANOVA**				
Decade	-	3, 470	48.4	**<0.001**
Species	-	3, 470	0.792	0.499
Season	-	9, 1416	6.25	**<0.001**
Decade * Species	-	3, 470	0.294	0.830
Species * Season	-	9, 1416	3.96	**<0.001**
Decade * Season	-	9, 1416	2.34	**0.013**
Decade * Species * Season	-	9, 1416	3.63	**<0.001**
**ANOVA C:N**				
Decade	1.97	1	<0.001	0.956
Season	1.12	3	13.4	**<0.001**
Species * Season	0.072	3	0.860	0.463
Decade * Season	0.134	3	1.61	0.186
Decade * Species * Season	0.018	3	0.210	0.890
**ANOVA N:P**				
Decade	1.92	1	93.4	**<0.001**
Season	0.06	3	0.930	0.427
Species * Season	0.013	3	0.210	0.890
Decade * Season	0.049	3	0.800	0.493
Decade * Species * Season	0.005	3	0.080	0.973
**ANOVA C:P**				
Decade	1.97	1	44.9	**<0.001**
Season	1.65	3	12.5	**<0.001**
Species * Season	0.097	3	0.740	0.528
Decade * Season	0.284	3	2.16	0.092
Decade * Species * Season	0.008	3	0.060	0.981

Supplementary Table 3. *Sargassum* tissue nutrient contents by decade and location.
Tissue nutrient contents (mean ± SE) of *Sargassum natans* and *S. fluitans* collected throughout the North Atlantic Ocean showing the stable N ($\delta^{15}N$) isotope values, elemental composition (%C, %N, %P), and stoichiometry (C:N:P) by decade and location.

Decade	Ocean Basin	Site	n	δ15N (‰)	%C	%N	%P	C:N	C:P	N:P
**1980s**			**41**		**23.1 ± 0.59**	**0.89 ± 0.06**	**0.18 ± 0.017**	**36.1 ± 2.92**	**497 ± 54**	**13.2 ± 0.8**
	North Atlantic		*39*		*23.3 ± 0.60*	*0.90 ± 0.06*	*0.18 ± 0.018*	*36.4 ± 3.06*	*506 ± 56*	*13.3 ± 0.8*
		Looe Key	*10*		*25.8 ± 0.49*	*1.09 ± 0.16*	*0.24 ± 0.041*	*34.6 ± 5.79*	*402 ± 89*	*11.2 ± 1.2*
		Neritic Baseline	*14*		*22.8 ± 0.77*	*1.05 ± 0.07*	*0.24 ± 0.019*	*26.6 ± 1.80*	*264 ± 26*	*9.8 ± 0.5*
		Oceanic Baseline	*15*		*22.1 ± 1.23*	*0.65 ± 0.07*	*0.08 ± 0.010*	*46.7 ± 5.90*	*802 ± 87*	*18.0 ± 1.1*
	Western Caribbean	Neritic Baseline	*2*		*18.8 ± 0.35*	*0.72 ± 0.01*	*0.16 ± 0.005*	*30.0 ± 1.00*	*311 ± 5*	*10.4 ± 0.4*
**2010s**			**447**	**1.55 ± 0.12**	**28.5 ± 0.14**	**1.21 ± 0.02**	**0.10 ± 0.002**	**32.4 ± 0.72**	**886 ± 24**	**27.8 ± 0.5**
	Eastern Caribbean		*10*	*-0.24 ± 0.24*	*27.4 ± 0.79*	*0.97 ± 0.08*	*0.12 ± 0.012*	*35.6 ± 3.69*	*676 ± 128*	*19.5 ± 2.5*
		Barbados	4	0.41 ± 0.22	29.1 ± 0.27	0.74 ± 0.03	0.13 ± 0.021	46.2 ± 1.76	595 ± 76	13.0 ± 1.8
		Saba	1	-1.83	31.6	0.78	0.05	47.2	1787	38.0
		St. Thomas	5	-0.43 ± 0.14	25.1 ± 0.26	1.20 ± 0.06	0.13 ± 0.006	24.9 ± 1.36	518 ± 23	20.9 ± 0.7
	Gulf of Mexico		*264*	*2.05 ± 0.18*	*28.6 ± 0.19*	*1.19 ± 0.03*	*0.10 ± 0.003*	*33.1 ± 0.92*	*944 ± 31*	*29.1 ± 0.6*
		GOM-AL-SUM	14	0.87 ± 0.11	26.1 ± 0.51	1.17 ± 0.10	0.09 ± 0.008	29.1 ± 2.86	846 ± 86	29.4 ± 1.1
		GOM-FE	33	-0.05 ± 0.10	30.1 ± 0.18	1.16 ± 0.04	0.08 ± 0.004	31.9 ± 1.18	1026 ± 49	32.3 ± 1.1
		GOM-FL-SUM	3	5.47 ± 0.20	24.5 ± 0.18	2.55 ± 0.15	0.21 ± 0.003	11.3 ± 0.76	297 ± 5	26.4 ± 1.5
		GOM-LA-FALL	20		33.4 ± 0.45	0.79 ± 0.07	0.06 ± 0.006	57.0 ± 4.87	1823 ± 197	31.2 ± 1.5
		GOM-LA-SUM	94	1.01 ± 0.13	27.7 ± 0.18	1.01 ± 0.03	0.09 ± 0.002	35.4 ± 1.20	884 ± 29	26.4 ± 0.9
		GOM-SHELF-SUM	40	-0.23 ± 0.08	26.4 ± 0.29	1.13 ± 0.03	0.10 ± 0.004	28.5 ± 1.12	762 ± 52	26.2 ± 0.9
		GOM-TX-SPR	18	7.20 ± 0.27	27.2 ± 0.71	1.75 ± 0.07	0.16 ± 0.013	18.5 ± 0.82	491 ± 46	25.9 ± 1.6
		GOM-TX-SUM	24	5.01 ± 0.27	30.6 ± 1.12	0.99 ± 0.09	0.07 ± 0.005	42.6 ± 3.58	1306 ± 94	33.0 ± 2.2
		GOM-TX-WIN	18	7.59 ± 0.19	30.8 ± 0.41	2.28 ± 0.10	0.15 ± 0.018	16.4 ± 0.78	686 ± 91	39.6 ± 3.5
	North Atlantic		*163*	*1.02 ± 0.12*	*28.3 ± 0.18*	*1.27 ± 0.03*	*0.12 ± 0.004*	*29.7 ± 1.00*	*768 ± 37*	*26.1 ± 0.9*
		Abacos	6	-1.28 ± 0.21	26.5 ± 1.12	1.17 ± 0.05	0.10 ± 0.006	26.7 ± 1.28	716 ± 18	27.2 ± 1.5
		Amazon River Plume	6	0.91 ± 0.83	29.1 ± 0.65	1.25 ± 0.38	0.11 ± 0.026	39.8 ± 8.48	823 ± 148	22.6 ± 2.3
		Biscayne_GS	4	-0.63 ± 0.10	30.4 ± 0.46	0.89 ± 0.03	0.09 ± 0.002	40.2 ± 1.44	856 ± 27	21.4 ± 0.8
		Fort Pierce_GS	5	-0.44 ± 0.11	28.2 ± 0.49	1.11 ± 0.14	0.10 ± 0.012	31.8 ± 4.18	737 ± 85	23.4 ± 0.8
		Fort Pierce_NS	15	2.78 ± 0.24	28.1 ± 0.42	1.42 ± 0.05	0.14 ± 0.004	23.5 ± 0.73	526 ± 20	22.7 ± 1.1
		Islamorada_GS	3	-0.39 ± 0.18	30.5 ± 0.17	0.69 ± 0.03	0.06 ± 0.004	52.0 ± 2.42	1326 ± 75	25.6 ± 1.5
		Key West	3	0.16 ± 0.15	31.9 ± 0.76	0.61 ± 0.05	0.05 ± 0.005	62.4 ± 6.83	1784 ± 193	28.7 ± 1.5
		Looe Key	84	0.97 ± 0.15	28.0 ± 0.25	1.30 ± 0.04	0.13 ± 0.005	28.2 ± 1.28	679 ± 38	24.5 ± 1.0
		Miami	3	1.42 ± 0.19	23.3 ± 0.21	1.82 ± 0.06	0.15 ± 0.004	15.0 ± 0.52	393 ± 11	26.3 ± 0.6
		Munson Island	16	0.18 ± 0.20	28.0 ± 0.41	1.01 ± 0.07	0.08 ± 0.007	35.7 ± 3.12	1120 ± 189	29.9 ± 2.5
		New Smyrna Beach_GS	3	-0.56 ± 0.09	29.6 ± 0.11	0.81 ± 0.04	0.08 ± 0.001	42.7 ± 2.13	964 ± 21	22.7 ± 0.7
		Savannah_GS	6	2.50 ± 0.29	27.7 ± 0.49	1.84 ± 0.06	0.17 ± 0.008	17.8 ± 0.87	428 ± 26	24.2 ± 0.9
		Sebastian Inlet	6	3.44 ± 0.23	30.5 ± 0.85	1.73 ± 0.11	0.16 ± 0.006	21.0 ± 1.49	511 ± 33	24.7 ± 1.5
		W. Florida Bay	3	3.23 ± 0.19	29.8 ± 0.65	1.33 ± 0.06	0.03 ± 0.001	26.3 ± 1.41	2337 ± 125	89.4 ± 5.0
	Western Caribbean		*10*	*-0.24 ± 0.30*	*31.5 ± 0.72*	*0.83 ± 0.08*	*0.06 ± 0.003*	*53.5 ± 11.2*	*1487 ± 126*	*32.1 ± 2.9*
		Belize	4	0.78 ± 0.26	34.0 ± 0.36	0.70 ± 0.17	0.05 ± 0.004	74.6 ± 25.8	1876 ± 146	32.5 ± 7.0
		Jamaica	6	-0.91 ± 0.13	29.9 ± 0.39	0.91 ± 0.06	0.06 ± 0.003	39.5 ± 2.87	1228 ± 72	31.8 ± 2.3
**Grand Total**			**488**	**1.55 ± 0.11**	**28.0 ± 0.15**	**1.18 ± 0.02**	**0.11 ± 0.003**	**32.7 ± 0.71**	**853 ± 23**	**26.6 ± 0.5**

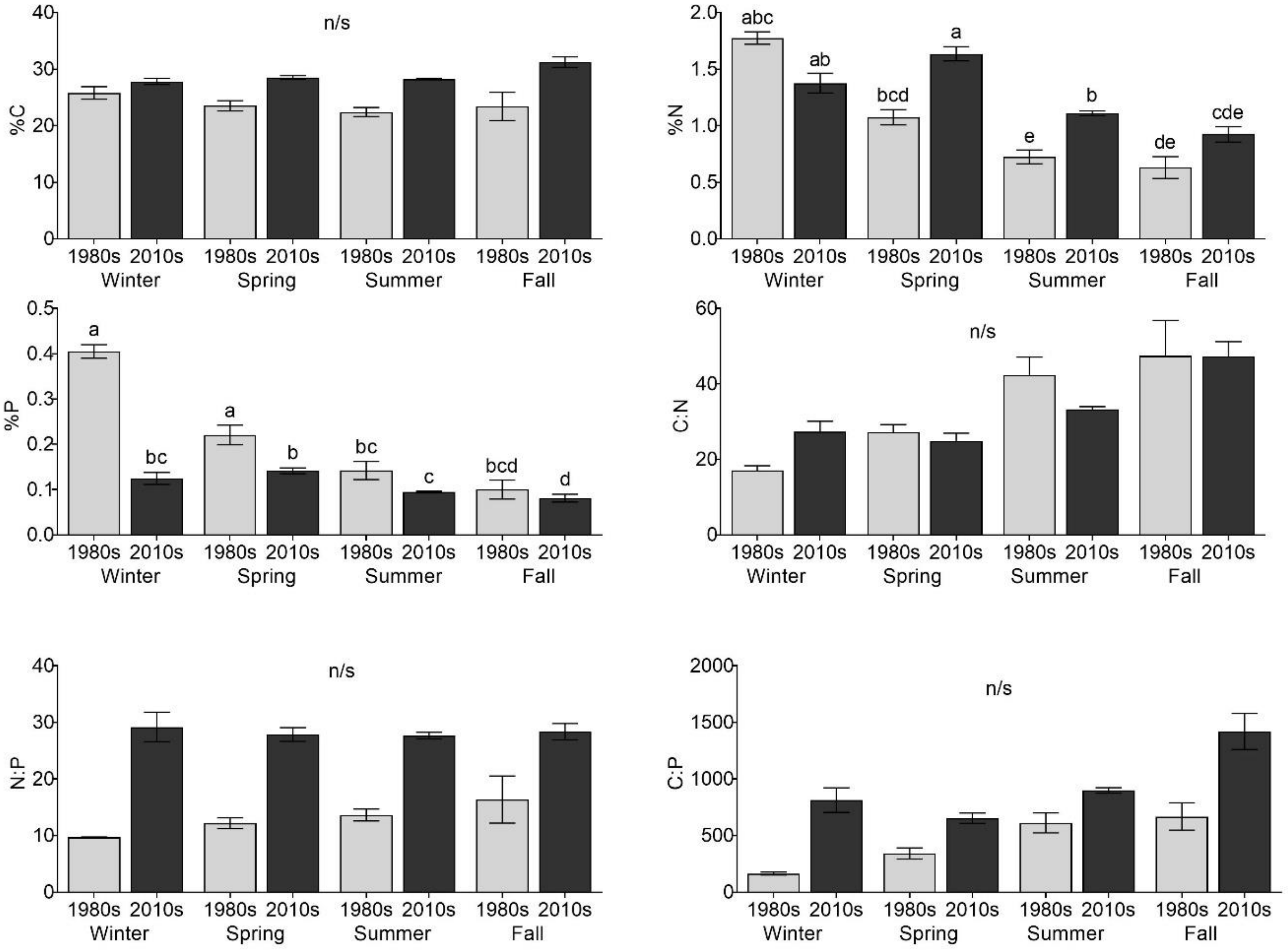

Supplementary Fig. 1: *Sargassum* tissue nutrient contents by season and decade.
Tissue elemental composition and stoichiometry (mean ± SE) of *Sargassum natans* and *S. fluitans* collected throughout the North Atlantic Ocean showing the interaction of decade and Northern Hemisphere meteorological season with different lowercase letters representing significant differences identified with Tukey HSD test; “n/s” denotes a non-significant ($P > 0.05$) ANOVA result.

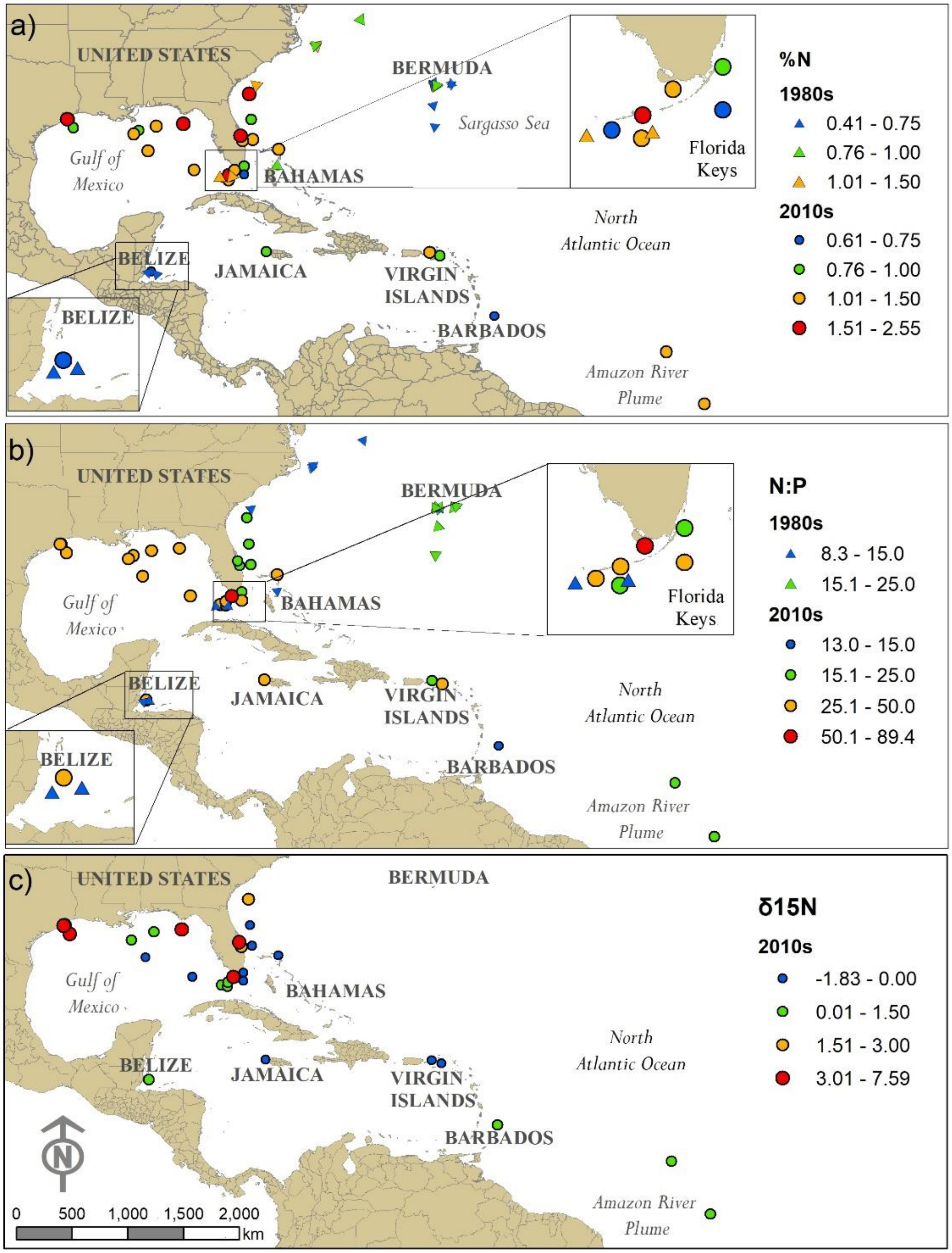

Supplementary Fig. 2: *Sargassum* nutrient contents by location and decade. *Sargassum* tissue nutrient contents by location and decade (showing location means). a) For %N, values have significantly increased from the 1980s (decadal mean = 0.89%) to post-2010 (decadal mean = 1.21%); > 1.5% N (red circles) are considered non-limiting to growth[1]. N:P ratios have significantly (111%; ANOVA, F = 1 93.4, P < 0.001) increased from the 1980s (decadal mean = 13.2) to post-2010 (27.8). Enriched $\delta^{15}N$ values (red circles; >3‰) are indicative of urbanized wastewater discharges; while more depleted values (blue, green, and orange circles) are indicative of N_2 fixation, atmospheric deposition, and upwelling.

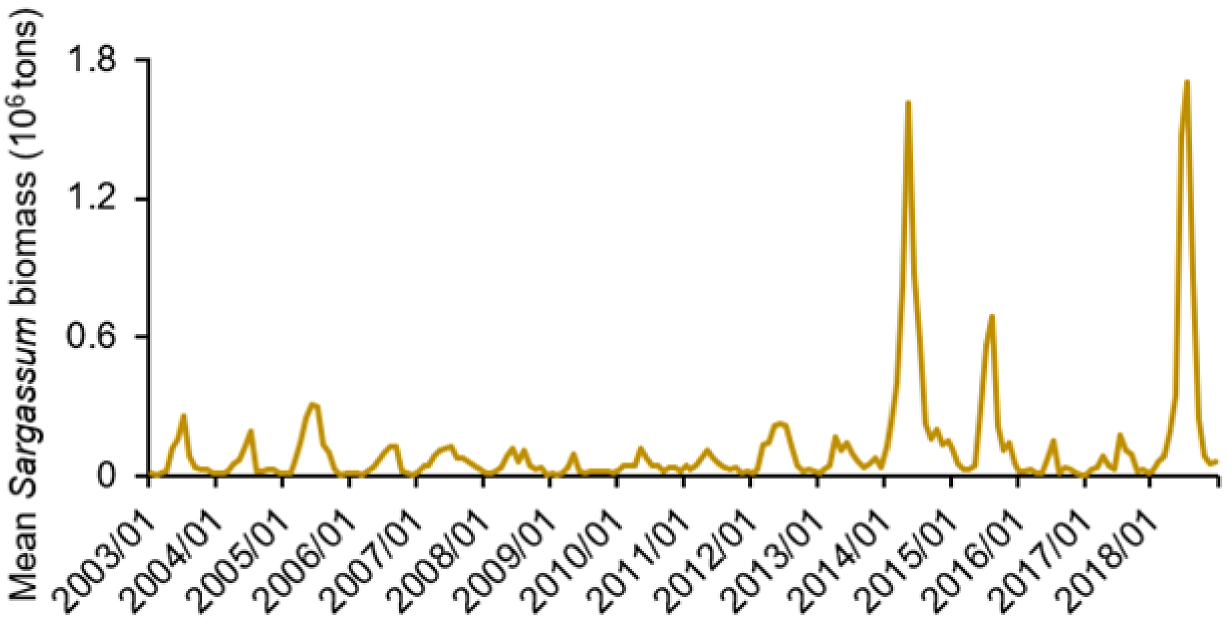

Supplementary Fig. 3: *Sargassum* monthly biomass. Monthly mean *Sargassum* wet biomass in the GOM (18ºN – 32ºN, 98ºW – 80ºW), determined from satellite measurements [2,3].

References

1. Fujita, R., Wheeler, P. & Edwards, R. Assessment of macroalgal nitrogen limitation in a seasonal upwelling region. *Mar. Ecol. Prog. Ser.* **53**, 293-303 (1989).
2. Wang, M. *et al.* Remote Sensing of Sargassum Biomass, Nutrients, and Pigments. *Geophys. Res. Lett.* **45**, 12,359-312,367 (2018).
3. Wang, M. *et al.* The great Atlantic *Sargassum* belt. *Science* **364**, 83-87 (2019).
