# Supplementary material for: Nutrient content and stoichiometry of pelagic Sargassum reflects increasing nitrogen availability in the Atlantic Basin
Source: Nat Commun. 2021 May 24;12:3060. doi: 10.1038/s41467-021-23135-7 (PMC8144625; doi:10.1038/s41467-021-23135-7)
Supplement: Supplementary file 4 — Description of Additional Supplementary Files [file 41467_2021_23135_MOESM4_ESM.docx]

Description of additional supplementary information

Title: Supplementary Video

Description: Video of Sargassum blooms
